# Parkinson’s Disease and the Cardiac Cycle: A Rapid Literature Review and Case Series

**DOI:** 10.3390/life13041003

**Published:** 2023-04-13

**Authors:** Holly Bardutz, Jyotpal Singh, Ziaur Rehman, Patrick Bernat

**Affiliations:** 1 Faculty of Kinesiology & Health Studies, University of Regina, Regina, SK S4S 0A2, Canada; 2 Department of Medicine, University of Saskatchewan, Regina, SK S4P 0W5, Canada

**Keywords:** Parkinson’s Disease, cardiac cycle, systolic function, diastolic function

## Abstract

Background and Objectives: Cardiac function in patients with Parkinson’s Disease (PD) is not well understood. We conducted a review of the literature to summarize all available data on the cardiac cycle in patients with PD and followed up the review with a case series to describe the cardiac cycle timing intervals in this patient population. Methods: Using the search terms (“Cardiac cycle” OR “echocardiography” OR “LVET” OR “IVCT” OR “IVRT” OR “LVEF” OR “Systolic Dysfunction” OR “Diastolic Dysfunction”) AND (“Parkinson’s Disease”), 514 studies were found with 19 included in the review. Results: Studies focused on the impact of medication, the presence of autonomic dysfunction, and resting-state, descriptive observational studies which considered the cardiac cycle. While inconsistent, the evidence suggests that patients with PD have some systolic dysfunction, with recent research implying the presence of subclinical systolic dysfunction. From the case series, 13 participants with PD were included and collected cardiac data daily for 6 weeks. Heart rate was consistent on a weekly basis at 67–71 bpm. Mean week-by-week cardiac parameters were also consistent with the systolic time interval at 332–348 ms, isovolumic relaxation time at 92–96 ms, and isovolumic contraction time at 34–36 ms. Conclusion: These timing intervals add valuable normative values for this patient population and the review of the literature suggests that more research is required to better understand cardiac cycle timing intervals in patients with Parkinson’s Disease.

## 1. Introduction

Parkinson’s Disease (PD) is prevalent in 0.2% of private households and 4.9% of residential areas in Canada [1]. Pharmaceutical interventions and exercise have gained interest and research, garnering these approaches as potential therapeutics. Recently, there has been growing evidence of impairments in sleep function in patients with PD as compared to controls [2]. From 157 patients with PD, with 95 at the early stage and 62 with late-stage PD, it was found that both groups had impaired sleep parameters, including sleep efficiency, wake after sleep onset and total sleep time as compared to controls when measured by actigraphy [3].

While sleep is better understood now in PD, there is still a need to understand how cardiac function is affected in PD. Indeed, using echocardiography, PD patients have shown reduced global longitudinal strain and ejection fraction, along with elevated left ventricular end-diastolic and systolic dimensions [4]. The authors state there does appear to be subclinical left ventricular systolic dysfunction in PD patients, which does correlate with cognitive impairment [4]. Heart rate variability (HRV) is a commonly used technique for the assessment of autonomic function, with depressed HRV in patients with PD [5]. However, HRV is generally associated with measures of sympathetic and parasympathetic tone [6], with the findings more consistent with inflammation and stress [6,7], and can be limited when attempting to understand the cardiac cycle. To understand the mechanism associated with cardiac changes in PD, there must be an emphasis on understanding changes associated with the cardiac cycle timing intervals. We conducted a literature review on the cardiac cycle parameters in PD patients and provided a proof-of-concept case series describing the cardiac cycle in a cohort of both exercising and non-exercising PD patients.

## 2. Materials and Methods

### 2.1. Literature Review

**Figure 1:**
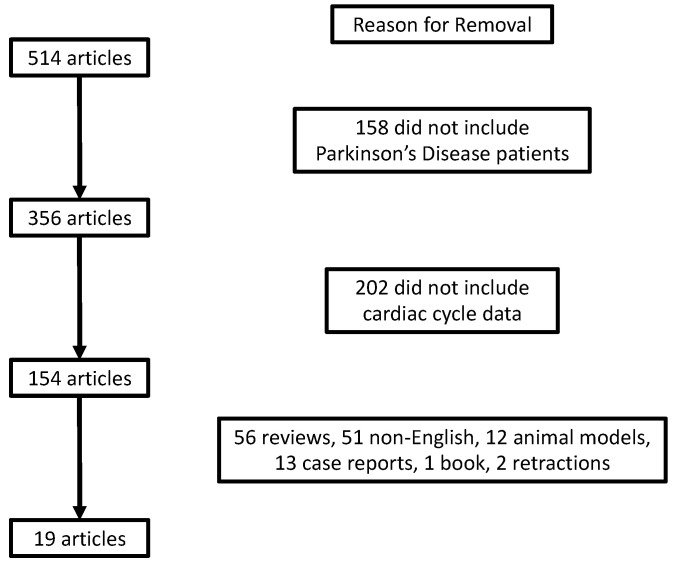
Flowchart of article exclusion for review.

A literature review was conducted to understand the findings of cardiac cycle intervals in PD. PubMed (including MEDLINE) was searched from the first available article to June 1, 2022, using the search terms (“Cardiac cycle” OR “echocardiography” OR “LVET” OR “IVCT” OR “IVRT” OR “LVEF” OR “Systolic Dysfunction” OR “Diastolic Dysfunction”) AND (“Parkinson’s Disease”) for peer-reviewed studies published in English. This search yielded 514 articles. After the removal of articles that did not include PD patients, 356 remained. Two-hundred and two articles that did not include cardiac cycle data, and 135 articles that were either not in English, or did not include humans, case reports, reviews, books, or retractions were also removed. This resulted in 19 articles that were included in our analyses (Figure 1).

### 2.2. Case Series Methods

This study was approved by the University of Regina research ethics board (REB-2020-152). Eighteen participants were recruited. All participants read and signed an informed consent form prior to beginning the study. Participants completed a Montreal Cognitive Assessment (MoCA) and were required to score ≥23 to proceed with the study. Participants were given an actigraph (Phillips Respironics, Actiwatch) and wore the watch at least 1 h before going to sleep and for 1 h after getting out of bed. Participants were given a cardiac sensor (Recordis^TM^, LLA Technologies, Langley, BC, Canada) and collected their cardiac data for 1 min every morning. Participants were told to rest in a supine position for 1 min and placed the sensor on their sternum. The sensor was turned on for 1 min and recorded the cardiac vibrations [8,9,10,11]. Data were collected for 6 weeks. Exercisers completed a minimum of 150 min/week of exercise on their own or by involvement in an online exercise program. Sleep data were assessed using the Phillips Actiware software. Their algorithm allows for the computation of sleep metrics, with our focus being sleep efficiency, calculated as the time spent sleeping/total time spent in bed.

As stated by [8,9,10,11], data analysis of the fiducial points of the cardiac cycle included the mitral valve closure (MVC), aortic valve opening (AVO), aortic valve closure (AVC), mitral valve opening (MVO), and twist force (TF). After the extraction of morphological features, temporal features were calculated (milliseconds, ms). This included diastole (MVC—MVO timing), systole (AVO—AVC timing), isovolumic contraction time [IVCT (MVC—AVO)], isovolumic relaxation time [IVRT (AVC—MVO)] and heart rate (AVOn + 1—AVOn). Performance indices were calculated as [12]:

- Diastolic performance index: DPI = IVRT/Ejection time
- Systolic performance index: SPI = IVCT/Ejection time
- Heart (or myocardial index) performance index: HPI (or MPI) = (IVCT + IVRT)/Ejection time

Due to the small sample size, descriptive statistics are presented as mean ± standard deviation. Percent (%) changes show differences in exercisers compared to non-exercisers on a week-by-week basis.

## 3. Results

### 3.1. Review Results

The findings of the studies included in the review are summarized in Table 1.

#### 3.1.1. Baseline Observational Studies

Using 2D speckle tracking echocardiography, decreases in LVEF and global longitudinal strain and increases in left ventricular end-diastolic and systolic dimensions were found in patients with PD as compared to controls under resting conditions [4]. These speckle-tracking strain changes imply that subtle myocardial deformations and subclinical systolic dysfunction may be present in patients with PD. Another study observed cardiac changes against healthy controls and found elevated stroke volume index, ventricular and atrial mass, and filling pressures as defined by the E/e’ ratio. Furthermore, these findings implied a greater prevalence of diastolic dysfunction and with the MPI at 0.4, there is a suggestion of altered timing intervals (IVRT, IVCT and ejection time) for the PD patients [13]. Only one study assessed atrial conduction times, with results suggesting that PD patients exhibit prolonged interatrial, and right and left intra-atrial electromechanical delay, and this prolongation is associated with disease severity [14]. These prolonged atrial conduction timing intervals can further imply that left ventricular intervals may also be prolonged.

#### 3.1.2. Autonomic Dysfunction

Utilizing 2D transthoracic echocardiography, it was found that PD patients with difficulties associated with blood pressure regulation (“reverse dipping”) have altered cardiac function in comparison to those with hypertension, as shown by PD patients having increased LV volume, LV mass, signs of LV hypertrophy, and signs of decreased LV filling pressure [15]. Building on autonomic dysfunction in PD patients, separating patients into those with a vasodilation or vasoconstriction response to a 70-degree head up tilt test found that the PD patients with a vasodilation response showed elevated total peripheral resistance and lower stroke volume and cardiac output when compared to vasoconstrictors and healthy controls [16]. In PD patients without neurogenic orthostatic hypotension, no altered cardiac cycle intervals were found to be associated with any PD severity demographics [17], and PD patients who had suffered from HF showed no differences in LVEF compared to PD patients who did not [18].

#### 3.1.3. Impact of Medication

When comparing PD patients on different medications given in their early or late-stage PD, one study found that when assessing cardiac function using speckle-tracking echocardiography, there are no differences between any of the groups when observing strain parameters [19]. Indeed, drug-naïve PD patients showed no differences in echocardiography parameters when compared to healthy controls [20].

Limited research is available on interventions and the cardiac cycle; however, of the available data, exercise training such as coordination and manipulation therapy has been shown to increase LVEF in PD patients at 6 and 12-month follow-ups [21]. Another intervention observed cardiac changes in PD patients undergoing L-dopa/dopa decarboxylase inhibitor therapy in comparison to healthy controls, with results showing no differences in strain parameters or LVEF [22]. However, a decrease in the E/A ratio, a common marker of LV diastolic function, was found in PD patients [22]. An observational assessment of PD patients using cabergoline found that the E/A ratio and deceleration time of the ventricle is associated with cumulative cabergoline dose [23]. While ergot-derived dopamine agonists are known to induce a risk of valvular dysfunction, no differences were seen in the cardiac cycle parameters, including strain parameters or LVEF, thus implying that there are no differences in myocardial systolic or diastolic function between groups [24]. Even when treated with ergot-derived dopamine agonists for 6 months, no differences in LVEF are prominent [25]. The use of pergolide or cabergoline also does not seem to alter LVEF [26]. The systolic time interval has been shown to be elevated in PD patients following a stimulus such as an exercise in comparison to healthy, younger subjects (20 ± 2 years), and altered pre-ejection time period dynamics were found in PD in multiple studies [27,28], including after tyramine injection [29] and Levodopa therapy [30].

Given these findings, there is a requirement for research focusing on describing the cardiac cycle intervals in PD patients. Furthermore, these cardiac studies do not include any information on the PD patients’ sleep quality.

**Table 1:** Summary of Cardiac Cycle findings in Parkinson’s Disease.

Author	Population	Primary Method of Assessment	Primary Cardiac Findings
(Erken Pamukcu et al., 2018) [19]	40 Parkinson’s Disease (H&Y = 2.5) and 40 healthy controls	Conventional and strain echocardiography	Reduced global longitudinal strain, left ventricular end-diastolic dimension, left ventricular end-systolic dimension and ejection fraction
(Strano et al., 2016) [20]	18 Parkinson’s Disease (H&Y = 1 or 2) and 18 healthy controls	Transthoracic echocardiography	No differences between groups
(Zhao et al., 2017) [21]	22 Parkinson’s Disease in coordination and manipulation therapy and 14 Parkinson’s Disease control PD (H&Y = 2–4)	Not stated	Baseline LVEF was lower in the coordination and manipulation therapy group, and increased over time
(Gunaydin et al., 2016) [22]	55 Parkinson’s Disease patients with L-dopa therapy and 30 healthy controls (H&Y = 1–4)	Transthoracic and strain echocardiography	Reduced E/A in PD, however, Levodopa therapy has no impact on left ventricular systolic function
(Ozer et al., 2014) [23]	34 Parkinson’s Disease cabergoline and 42 Parkinson’s Disease no cabergoline	Conventional echocardiography and tissue doppler imaging	Only diastolic differences (E parameters) associated with cumulative cabergoline dose
(Rasmussen et al., 2008a) [24]	71 Parkinson’s Disease with EDDA for at least 6 months and 39 non-EDDA	Conventional and speckle tracking echocardiography and tissue doppler imaging	No differences in myocardial systolic and diastolic function between groups
(Rasmussen et al., 2008b) [25]	85 Parkinson’s Disease (H&Y = 2.3) with EDAA and 53 non-EDAA (H&Y = 1.9)	Continuous wave Doppler	No differences in LVEF
(Kenangil et al., 2007) [26]	46 Parkinson’s Disease on pergolide or cabergoline or both for a minimum of 1 year and 49 healthy controls	Conventional echocardiography	No differences in LVEF
(Imrich et al., 2009) [27]	13 Parkinson’s Disease (H&Y = 1–5) or pure autonomic failure, 15 patients with multiple system atrophy and 5 healthy controls	Impedance cardiogram	Tyramine infusion had little response on cardiac intervals in PD
(Meijer et al., 2008) [28]	18 healthy young controls, 25 elderly healthy controls, 18 Parkinson’s Disease (H&Y = 1–3)	Impedance cardiogram	Reduced relative contribution of PEP to RR intervals and reduced exertion during exercise in PD
(Imrich et al., 2008) [29]	9 Parkinson’s Disease and 3 with pure autonomic failure	Impedance cardiogram	No reduction in PEP following tyramine infusion
(Whitsett & Goldberg, 1972) [30]	6 Parkinson’s Disease assessed after 2 weeks of levodopa therapy and after 3 months of continuous therapy	Carotid pulse contour and phonocardiogram	PEP responses of acute and chronic levodopa to dopamine and epinephrine intake are not different

H&Y: Hoehn and Yahr; LVEF: Left ventricular ejection fraction; PEP: Pre-ejection period.

### 3.2. Case Series Results

Of the 18 participants recruited, 13 completed the 6-week data collection. The demographics are presented in Table 2.

Heart rate was consistent on a weekly basis at 67–71 bpm. Mean week-by-week cardiac parameters were also consistent with systole at 332–348 ms, IVRT at 92–96 ms, IVCT at 34–36 ms, SPI at 0.10–0.11, DPI at 0.27–0.29, HPI (MPI) at 0.37–0.40, and cardiac TF at 12–14 mG.

Six exercisers and seven non-exercisers were included. Weekly means and % changes for cardiac parameters are presented in Table 3. Cardiac parameters were consistent for exercisers with systole at 342–353 ms, IVRT at 94–98 ms, IVCT at 34–37 ms, SPI at 0.10–0.11, DPI at 0.28 to 0.29, HPI at 0.38–0.40, and TF at 11–13 mG on a weekly basis.

For non-exercisers, cardiac parameters were also consistent week-by-week with systole at 321–348 ms, IVRT at 89–94 ms, IVCT at 33–35 ms, SPI at 0.10–0.11, DPI at 0.27 to 0.30, HPI at 0.36–0.42, and TF at 13–17 mG. Timing parameters for exercisers show between 3.8 and 6.2% changes in IVRT, −0.4 and 11.8% changes in IVCT, and −1.7 and 6.6% for systole. Performance indices ranged from −0.4 to 11.0% changes in SPI, −7.9 to 8.7% changes in DPI, and −6.8 to 9.5 % changes in HPI (MPI). Contractile parameter TF % changes ranged from −7.6 to −23.2%.

Of the secondary measures, diastole ranged from 411 to 449 ms per week for non-exercisers and 405–430 ms per week for exercisers. These changes represent a 1.6–6.3% difference per week between exercisers and non-exercisers. Heart rate ranged from 67 to 72 bpm per week for non-exercisers and 68 to 70 bpm for exercisers. These changes represent a 0.1–5.7% difference per week between exercisers and non-exercisers.

Sleep data showed that changes in sleep efficiency were minimal between exercisers and non-exercisers. Sleep efficiency ranged from 76 to 81% per week in non-exercisers and 80 to 85% per week in exercisers. % Changes in sleep efficiency ranged from 0.6 to 6.8% per week in exercisers compared to non-exercisers.

## 4. Discussion

In comparison to healthy normative data available in the literature, these descriptive results suggest that PD patients have a prolonged systolic time interval. Stratifying by exercisers and non-exercisers shows similar results between the groups. Sleep efficiency was also consistent between groups.

Cardiac function is not well studied in PD. As shown by the literature review, there is evidence for subclinical dysfunction, implying reduced LV function [4], and further timing deficits [14] imply that there may be cardiac cycle impairments in patients with PD.

Cardiac timing intervals and performance indices have been reported for healthy individuals in comparison to those with a history of cardiac complications [31]. In healthy individuals, there are differences based on sex and age [31]. In comparison to the healthy data in the literature, our group shows increased systole from the healthy 279 ms for females and 289 ms for males. The participants in this study exhibited a systolic time interval of 332–348 ms, thereby resulting in reduced performance indices. Elevations in HPI and DPI are associated with coronary heart disease and a history of major adverse cardiac events [12,32]. Our results show that the decreased HPI in PD patients can be due to the increase in the systolic time interval. Similar to research available in the literature on systolic dysfunction, our results imply that there may be a prolonged AVO period, resulting in elevated systolic time in patients with PD. However, we did not have a direct age-matched comparison group.

Our results show that sleep efficiency is consistently around 80% in PD patients. Sleep is known to be strongly affected by PD; however, exercise seems to aid in helping to reduce some of these sleep impairments [33]. A randomized, controlled trial showed improvements in sleep efficiency and sleep architecture in PD patients undergoing supervised resistance training exercise three times per week for sixteen weeks, including time spent SWS and total sleep time, as compared to a group in sleep hygiene being given advice on sleep [34]. Considering that our data showed consistent sleep efficiency results, there may be a potential influence from non-exercisers being active, even if they do not engage in organized exercise programs. Therefore, monitoring sleep function can be vital when assessing physiology in patients with PD.

Limitations of this study include the current sample size, self-statements for stratification of exercisers and non-exercisers, and exclusion of data for quality control. Furthermore, the non-exercisers completed general daily activities and the exercisers varied in exercise intensity and quantity. More randomized controlled trials are needed to understand the cardiac cycle in patients with PD. These can include more exercise studies to understand how exercise can influence the cardiac cycle, and studies that include a focus on how medications can influence the cardiac cycle.

## 5. Conclusions

The literature review suggests that there are alterations in systolic function in patients with PD; however, this finding is not always consistent. Recent research suggests that subclinical systolic dysfunction may be prevalent in patients with PD; however, more research is required to validate these findings. What is not well understood in the literature are the cardiac cycle timing intervals. The case series described cardiac parameters which include systole, IVCT, IVRT, HPI, DPI and SPI, and compared those between exercising and non-exercising PD patients. The normative data presented provide insight and comparable values that can be of clinical utility. More participants assessed over a greater number of weeks can provide further insights.

## Figures and Tables

**Table 2:** Demographic details of participants included in case series.

Participant Demographics	
Age (years) ± SD	70 ± 7
Weight (kg) ± SD	77 ± 12
Height (cm) ± SD	169 ± 7
BMI (kg/m^2^) ± SD	27 ± 5
Female (%)	33
% Hypertension	72
% Diabetes	28
% Other cardiac disease	72
% Prescribed Levodopa	100
% Exercisers *	46
Montreal Cognitive Assessment (mean ± SD)	26 ± 2

* Exercisers refers to those who complete at least 150 min/week of exercise/physical activity.

**Table 3:** Weekly changes in primary cardiac cycle parameters in exercisers and non-exercisers.

	W1	W1 EX	W2	W2 EX	W3	W3 EX	W4	W4 EX	W5	W5 EX	W6	W6 EX
IVRT (ms)	91 ± 7	97 ± 7	93 ± 8	98 ± 8	90 ± 5	97 ± 10	91 ± 4	98 ± 9	93 ± 8	98 ± 7	93 ± 3	100 ± 10
% Δ IVRT EX	4.4		6.2		5.2		3.8		3.9		4.8	
IVCT (ms)	34 ± 1	34 ± 2	33 ± 1	37 ± 3	33 ± 1	37 ± 3	33 ± 3	36 ± 2	35 ± 2	37 ± 2	35 ± 2	37 ± 1
% Δ IVCT EX	−0.4		9.5		9.3		11.8		3.9		5.4	
SPI	0.10 ± 0.01	0.10 ± 0.02	0.10 ± 0.02	0.11 ± 0.02	0.11 ± 0.03	0.11 ± 0.02	0.10 ± 0.02	0.11 ± 0.02	0.11 ± 0.03	0.11 ± 0.01	0.11 ± 0.02	0.11 ± 0.02
% Δ SPI EX	−0.4		11		−4		6.4		−2.9		2.6	
DPI	0.27 ± 0.02	0.28 ± 0.04	0.27 ± 0.03	0.29 ± 0.05	0.30 ± 0.09	0.28 ± 0.04	0.28 ± 0.03	0.29 ± 0.04	0.28 ± 0.03	0.28 ± 0.04	0.28 ± 0.04	0.29 ± 0.06
% Δ DPI EX	5.7		8.7		−7.9		2.1		−1.5		3.1	
HPI	0.37 ± 0.03	0.38 ± 0.06	0.36 ± 0.05	0.40 ± 0.06	0.42 ± 0.12	0.39 ± 0.05	0.38 ± 0.05	0.40 ± 0.05	0.39 ± 0.06	0.38 ± 0.05	0.39 ± 0.06	0.40 ± 0.07
% Δ HPI EX	4		9.5		−6.8		4.2		−2.4		2.9	
Systole (ms)	348 ± 43	347 ± 56	348 ± 53	342 ± 65	321 ± 56	342 ± 53	334 ± 33	343 ± 58	334 ± 50	353 ± 58	337 ± 43	347 ± 60
% Δ Systole EX	−0.2		−1.7		6.6		2.8		5.7		3.1	
TF (mG)	17 ± 6	13 ± 4	14 ± 2	12 ± 4	14 ± 2	11 ± 4	14 ± 4	11 ± 4	13 ± 3	12 ± 4	14 ± 3	12 ± 5
% Δ TF EX	−23.2		−18.3		−21.9		−23.2		−7.6		−12.6	
SE (%)	81 ± 11	83 ± 9	81 ± 9	84 ± 8	79 ± 13	81 ± 9	81 ± 12	82 ± 8	81 ± 12	84 ± 7	77 ± 15	82 ± 9
% Δ SE EX	2.3		2.6		3.0		0.8		3.7		6.0	

W#: Week number; EX: Exercise; IVRT: Isovolumic relaxation time; IVCT: Isovolumic contraction time; SPI: Systolic performance index; DPI: Diastolic performance index; HPI: Heart performance index; TF: Twist force; ms: millisecond; mG: milligravity; SE: Sleep Efficiency.

## Data Availability

The data presented in this study can be requested from the corresponding author following appropriate request.
